# The Effect of Social Isolation Types on Quality of Life during the Coronavirus Disease 2019 Pandemic in Saudi Arabia: A Cross-Sectional Study

**DOI:** 10.3390/ijerph19116808

**Published:** 2022-06-02

**Authors:** Aisha Alhofaian, Ruba Alharazi, Marym Alaamri, Samah Mahmoud Sofar, Afnan Tunsi, Mona Mohamed Elhady, Hayfa Almutary, Lujain Sallam, Shuruq Asiri

**Affiliations:** 1 Medical Surgical Nursing Department, Faculty of Nursing, King Abdulaziz University, P.O. Box 4929, Jeddah 22246, Saudi Arabia; aalhofaian@kau.edu.sa (A.A.); ralharazi@kau.edu.sa (R.A.); mmalamri@kau.edu.sa (M.A.); atunsi@kau.edu.sa (A.T.); lsallam@kau.edu.sa (L.S.); 2 Medical Surgical Nursing Department, Faculty of Nursing, Alexandria University, Alexandria 21544, Egypt; ssofar@kau.edu.sa; 3 Emergency and Critical Care Nursing, Faculty of Nursing, Mansoura University, Mansoura 35516, Egypt; mmmohamad@kau.edu.sa; 4 Critical Care Nursing Department, Faculty of Nursing, King Abdulaziz University, P.O. Box 4929, Jeddah 22246, Saudi Arabia; saaseere@kau.edu.sa

**Keywords:** COVID-19 pandemic, quality of life, Saudi Arabia, social isolation

## Abstract

COVID-19 has caused a global pandemic that has spread rapidly to six continents, with over 2.5 million deaths from December 2019 to March 2021. The number of confirmed COVID-19 cases is still growing worldwide, and quarantines have been recommended to prevent the disease’s spread. However, quarantines affect people’s quality of life (QOL). The aim of this study is to assess the effect of social isolation—quarantine—on QOL during the first wave of the COVID-19 pandemic in Saudi Arabia. A cross-sectional, descriptive design was used in the present study. We obtained data from an online survey conducted in Saudi Arabia. We included a convenience sample of 775 participants in the study. Most of the participants were female (67%), with a mean age of 46 years ranging from 18 to 75 years. Many participants were government employees (n = 308, 39.7%) and had a bachelor’s degree or greater (n = 513, 66.2%). Most of the participants (n = 629, 81%) were free from any chronic disease. Nearly 88% of participants were partially isolated socially owing to COVID-19. Concerning QOL, the means of both the Physical Health Composite Scale and the Mental Health Composite Scale SF-12 scores were 44.7 and 34.6, respectively. Furthermore, the results demonstrate that partially socially isolated participants appeared to have significantly better physical health statuses (M = 44.96, SD = 5.90) than completely socially isolated participants (M = 42.87 ± 7.26). There was no significant social isolation effect on mental health status (t (773) = 2.5, *p* = 0.115). Social isolation during the COVID-19 pandemic considerably influenced various aspects of QOL including physical and mental health. Community-based interventions such as online counseling services and wellness programs are required to reduce the pandemic’s negative impact and enhance overall health status and QOL.

## 1. Introduction

Coronavirus disease 2019 (COVID-19), a new strain of coronavirus identified in December 2019 in Wuhan City, Hubei Province of China, has spread rapidly throughout the world [1]. In March 2020, the World Health Organization (WHO) announced COVID-19 to be a pandemic [2]. As of 1 March 2021, COVID-19 had spread to six continents, affecting over 100 million people, and over 2.5 million people worldwide have died after contracting the respiratory virus [3]. In Saudi Arabia, the number of confirmed cases has exceeded 300,000 [4].

The number of confirmed COVID-19 cases is still growing, and the outbreak continues to progress worldwide [2]. The WHO recommends quarantine to prevent and control the spread of the disease to new regions and reduce its transmission [5,6]. To reduce the transmission of this communicable pandemic disease by respiratory droplets, and to control its outbreaks, several restrictions and preventive strategies have been implemented to reduce interactions between people, such as social isolation, physical distancing, travel restrictions, and closing of public places such as parks and gyms [2,7,8,9]. In Saudi Arabia, the government used social isolation and lockdown measures between February and June 2020 to control the spread of new coronavirus infections. There were two types of social isolation. Partial isolation was time-specific isolation from 6 am to 7 pm used for 21 days starting on 23 March 2020. Later, complete social isolation was the physical state of being entirely isolated from social environments and interaction patterns for the whole day, with the exception of one hour per day with permission, used in Riyadh, Dammam, Tabuk, Dahran, Hafuf, Jeddah, Taif, Qatif, and Khobar on 6 April 2020.

Social isolation is commonly defined as a lack of physical contact with other persons [10,11]. This is achieved by prohibiting gatherings, social contact with family and friends, and engagement in public activities; closing schools, workplaces, and fitness centers [9,12]; and limiting or closing public markets [13]. Under these circumstances, people have to stay at home and handle the difficulties of quarantine [7]. Fear of the disease and death; doubt, uncertainty, and anger; loss of access to social gatherings and outdoor activities; loneliness and helplessness; alterations in daily activities and routines; financial losses; and lack of food, water, or medical supplies can all act as stressors for people in quarantine [14].

Social isolation used for containing the COVID-19 pandemic has significant impacts on health, society, economic factors, and daily life routines [15]. Social isolation can also affect diet and exercise, leading to a decrease or increase in exercise and changes in sleep patterns and concentration abilities, thereby having negative and undesirable effects on health and quality of life (QOL) [12,16]. Social isolation can increase an individual’s risk of developing chronic health conditions [8]. Prolonged social isolation can lead to boredom and loneliness, which impacts mental and physical well-being [7,15].

QOL is a general sense of well-being that includes a feeling of satisfaction with life. QOL includes the psychological, physical, and social aspects of health that are affected by an individual’s beliefs, expectations, and experiences [17]. Governments navigated the COVID-19 pandemic through qfuarantines and imposed isolation, but these interventions can contribute to lower health-related quality of life (HRQoL) and higher rates of anxiety and depression [18,19,20]. Moreover, physical activity is vital for a good HRQoL [21] and has a major impact on anxiety and depression [22] because it helps in decreasing the risks of dysfunction, depression, anxiety, and cognitive impairment [23]. To help maintain QOL during social isolation, measures such as exercising regularly, eating a balanced and healthy diet, practicing relaxation exercises such as yoga and deep breathing, sleeping well and adequately, avoiding smoking, reducing the intake of negative television or social media news related to the pandemic, using time effectively, undertaking recreational activities, and communicating from a distance with others could be effective strategies for improving individual health and well-being during the pandemic [24].

Several studies across multiple countries have assessed the COVID-19 pandemic’s impact on HRQoL. Ferreira et al. (2021) [20] showed that isolated individuals in Portugal displayed decreased HRQoL and increased levels of anxiety, especially among women and the elderly. Differences between HRQoL and anxiety scores correlated with an individual’s employment status, marital status, place of residence, and religious beliefs, as well as whether they had a chronic illness, lived with vulnerable families, or supported other vulnerable people [25]. Meanwhile, Vitorino et al. (2020) investigated the effects of social isolation on quality of life (QoL) and mental health during the COVID-19 pandemic in Brazil [26]. They found an increased prevalence of symptoms related to depression (41.9%) and anxiety disorders among participants (29.0%). Negative psychological and religious coping was associated with anxiety and depression, as well as with poor environmental- and social-related quality of life [1]. However, when Zhang and Ma (2020) examined the quarantine’s effect on QoL and on mental health during the COVID-19 pandemic in China, they revealed that the quarantine only had a minor effect on the QoL of their participants [20].

Several studies have been conducted to investigate the clinical symptoms of COVID-19 [27,28] and its impact on mental health [1,7]. In addition, several researchers have investigated the relationship between social isolation and physical and mental health statuses [9,11,29,30,31,32]. However, to date, few scholars have investigated the effect of social isolation on the QOL of people in Saudi Arabia during the COVID-19 pandemic. Therefore, this study aimed to assess the effects of social isolation types on QOL in Saudi Arabia during the COVID-19 pandemic.

## 2. Methods

### 2.1. Design

We used a descriptive cross-sectional design to investigate the effects of enforced social isolation (quarantine) on health-related QOL during the COVID-19 pandemic in Saudi Arabia.

### 2.2. Settings

The targeted participants were recruited through an online survey platform (Google Forms). Online surveys were the most feasible method to reach the target population, considering the social distancing protocols implemented during the pandemic. To recruit participants, we circulated the online survey link through professional and social networks without identifiers, such as Twitter. We also shared the study invitation link through various online communication channels, including email, organizational portals, and social media platforms (WhatsApp, Twitter, and Facebook). Participants responded to the survey questions without identifiers.

### 2.3. Participants

The target population was adults aged 18 years and above of Saudi nationality or expatriates residing in Saudi Arabia during the COVID-19 pandemic period. We recruited participants using the convenience sampling method. We included participants of both sexes that had the ability to self-report by completing an anonymous online survey questionnaire. We estimated the sample size for this study as 600 participants, as calculated by n = (z)^2^ *p* (1 − *p*)/d^2^, with a 4% margin of error and a confidence level of 95%.

### 2.4. Measures

We used an online survey—a social-isolation-related QOL questionnaire—to collect data about the participants’ QOL during the quarantine period in the COVID-19 pandemic. The questionnaire comprised two parts: the participants’ demographic data and a health-related QOL survey (see Supplementary File).

#### 2.4.1. Participant’s Sociodemographic and Clinical Data Assessment

We obtained participants’ demographic data, including age, sex, education level, type of employment, social isolation status, and chronic illness, for descriptive purposes. By the time this study was conducted, the complete lockdown had been lifted in some regions; however, other regions, such as Mecca City, were still under enforced governmental lockdown. Hence, the social isolation status of the participants was either partial or complete. For the sake of clarity, the type of isolation status was defined in the questionnaire as follows: complete social isolation was the physical state of being entirely isolated from social environments and interaction patterns for the whole day, with the exception of one hour per day with permission, and partial isolation was time-specific isolation from 3 p.m. to 8 a.m. on the second day.

#### 2.4.2. Health-Related Quality of Life Survey

We measured the health-related QOL using the validated Arabic version of the Short Form (SF-12) Health Survey [33] developed by Ware et al. in 1998. The SF-12 is a valid generic measure of health outcomes used to examine QOL derived from the full version of the SF-36. It includes eight dimensions: physical functioning; role limitation due to physical health problems; body pain; general health; vitality; social functioning; role limitation due to emotional problems; and mental health. Ware et al. developed the scoring system of SF-12 [34]. We used the weighted means of the eight domains to calculate the summary scores of the SF-12—physical and mental health item of the questionnaire. These can range from 0 to 100, with 0 reflecting the lowest health level and 100 the highest level. We used the US norm-based scoring algorithm to compute the scoring of the SF-12 in Statistical Package for the Social Sciences (SPSS) software (IBM, Armonk, NY, USA) [34].

### 2.5. Patient and Public Involvement

Patients and the public were not involved in the design, conduct, reporting, or dissemination plans of our research.

### 2.6. Data Analysis

The data were analyzed using SPSS version 22. Descriptive statistics generated for demographics and study variables included measurements of central tendency (mean), variability (standard deviation), and distribution shape. One-way ANOVA and *t*-tests were used to examine differences between the means of groups, considering a *p*-value of ≤0.05 for statistical significance.

## 3. Results

Table 1 shows the sociodemographic and clinical characteristics of the participants. A total of 775 participants completed the questionnaires, indicating a response rate of 100%. There were no missing data. Most participants in this study were women (67%) and the mean age was 46 years. More than half of the participants had a college education. Most participants (n = 308, 39.7%) were employed by a government agency, whereas 36% were unemployed. Most participants (n = 629, 81%) were free from chronic diseases, whereas 18% of the participants had chronic diseases. The types of chronic diseases varied, but the most common type (n = 51, 35%) was diabetes, whereas the second most common type was asthma (n = 44, 30%). Approximately 88% of the participants (n = 682) were partially socially isolated owing to COVID-19.

Table 2 shows the descriptive statistics for the 12 questions from SF-12. The first item was “In general, would you say your health is?”, whose mean was 4.34, indicating that 86.8% of the participants were in excellent health. In terms of physical function, 83% of the participants declared that they could perform moderate activities without limitations. Furthermore, more than half of the participants said they did not find difficulty in climbing stairs. Whereas 24% said they could do less than they would have liked to do, 76% said they could do what they liked. In terms of physical pain, 79% stated that they experienced slight pain during everyday work. Of the activities that required movement, 40% felt they did less than what they would have liked to do. Nearly half of the participants were energetic.

Regarding social roles, more than half of the participants said that their health interfered with social activities, but only occasionally. We also calculated the summary scores of SF-12 for physical and mental health. According to the results, the mean Physical Health Composite Scale (PCS) score was 44.7 (range, 11 to 63) and the mean Mental Health Composite Scale (MCS) SF-12 score was 34.6 (range, 20 to 57). Lower scores indicated worse physical and mental health statuses.

Using an alpha level of 0.05, an independent-samples t-test was conducted to evaluate whether participants with different social isolation statuses (partial vs. complete) differed significantly in QOL. We found a statistically significant difference between social isolation status (partial vs. complete) and PCS scores. Specifically, partially socially isolated persons appeared to have better PCS scores (M = 44.96, SD = 5.90) than completely socially isolated persons (M = 42.87, SD = 7.26) (Table 3), whereas there was no significant effect of social isolation on MCS scores (t (773) = 2.5, *p* = 0.115) (Table 4).

In addition, the one-way ANOVA showed significant differences in MCS scores between the various age groups (*F* = 3.534, *p* ≤ 0.05). Participants aged 60 years and older (M = 37.73, SD = 8.34) showed better mental health than participants aged 31–39 (M = 34.07, SD = 8.40), but there were no significant differences in PCS scores between the various age groups (Table 5 and Table 6).

The *t*-test result showed no significant difference in MCS scores when considering the presence of chronic illnesses (*t* = 0.462, *p* > 0.05), but there was a significant difference in PCS scores. Participants without a chronic illness (M = 45.17, SD = 5.84) displayed better physical health compared to participants with a chronic illness (M = 42.70, SD = 7.01).

In addition, the *t*-test results showed no significant difference in MCS scores between male and female participants (*t* = 3.73, *p* > 0.05). Men (M = 36.29, SD = 8.71) displayed slightly better mental health compared to women (M = 33.88, SD = 8.27), but there were no significant differences in PCS scores between the two groups.

The one-way ANOVA demonstrated that the MCS scores differed based on area of employment (F = 5.187, *p* ≤ 0.05). Participants who worked in free business (M = 37.29, SD = 9.34) displayed better mental health than participants who worked in the private sector (M = 32.65, SD = 8.23) or in the government (M = 35.47, SD = 8.28). However, the analysis showed no significant differences in PCS scores with respect to area of employment, as reported in Table 5 and Table 6.

Finally, there are no statistically significant differences by the education level variable in either MCS or PCS scores, as reported in Table 5 and Table 6.

## 4. Discussion

Approximately 215 countries and territories worldwide have been affected by COVID-19. The virus still circulates at exceedingly high rates, and many countries have reintroduced lockdown rules to slow the spread recorded over the winter months. The continuously increasing number of COVID-19 cases worldwide causes stress, anxiety, and fear among the public [7]. It has a major impact on the QOL as well as the mental and physical health of individuals [9]. In this study, we aimed to assess the effect of social isolation on QOL in Saudi Arabia during the COVID-19 pandemic.

The overall health of the study participants was excellent, as reported by most respondents. In terms of physical function, most participants reported that they could perform moderate activities without limitations. More than half of the participants reported they did not find difficulty in climbing stairs. More than three quarters of the participants could do what they liked, whereas less than a quarter could do less than they would have liked to do. Regarding social roles, more than half of the participants said that their health interfered with social activities, but only occasionally.

This result contrasts those of Goethals et al. (2020), who reported that social distancing due to the COVID-19 pandemic could lead to negative consequences on the physical health of older adults [35]. Decreased physical activity levels are caused by the total or partial restriction of social participation in community groups and family activities during the pandemic. In addition, Güzel et al. (2020) reported that individuals staying at home because of COVID-19 stated that their lives were affected by anxiety and stress, their physical mobility was limited, and their physical activity levels decreased if they stayed at home.

In terms of physical pain, the present study revealed that more than three-quarters of the participants felt a small amount of pain during everyday work. Regarding activities that required movement, 40% of the participants felt they could do less than they would have liked to do. This result is similar to that of Lim et al. (2020), who found that nearly half of the participants had a great deal of energy [36]. Furthermore, nearly half of the participants (48.5%) reported problems in at least one dimension during the pre-pandemic period, with the majority reporting problems of pain or discomfort.

We also assessed physical and mental health using SF-12 summary scores. According to the survey results, the mean PCS and MCS SF-12 scores were 44.7 and 34.6, respectively. In addition, the PCS and MCS scores of the participants with chronic diseases were 42.70 ± 7.01 and 34.38 ± 9.15, respectively. These results align with those of Samlani et al. (2020), who reported that the SF-12 scores of people in quarantine were below the standardized mean of 50 and the participants with chronic diseases reported more negative physical and mental health statuses than the participants without chronic diseases [25].

In addition, our results align with those of Szczepanka and Pietrzyka (2021), who reported a negative impact of pandemic emergencies and lockdown rules on the daily activities of young adults in Poland (72%). The authors further disclosed deteriorating physical and psychological wellbeing as a result of social distancing, mainly due to the absence of human interactions [31].

Regarding the relationship between the QOL and social isolation status, the current study reveals a statistically significant difference in PCS scores based on social isolation status (partial vs. complete). Specifically, partially socially isolated persons appeared to have better PCS scores than completely socially isolated persons. Although there was no significant effect of social isolation on MCS scores, our results agree with those of Ferreira et al. (2021), which showed that isolated individuals in Portugal displayed decreased HRQoL and increased levels of anxiety, especially among women and the elderly [37]. This could mean that social isolation is not necessarily bad; family members may need to spend more time with each other, and isolation may provide them with this chance. In addition, most work was performed online to maintain mental health.

Furthermore, Pietrabissa and Simpson (2020) reported that prolonged isolation could adversely affect physical and emotional health, altering sleep and nutritional rhythms and reducing movement opportunities [21]. Moreover, Loyola et al. (2020) reported social participation to be associated with a better QOL and social distancing with reduced physical activity, which could harm physical health [18]. Finally, Reed et al. (2011) confirmed that isolated people have less physical activity and more sedentary behavior than those who are not isolated [19]. As in any study, there are some limitations in this study. This study was restricted to the Saudi population. Additionally, the use of a cross-sectional design meant that it could not assess the causality effect among the variables.

### Implication for Policy, Practice, and Research

The findings of this study have several implications for policy, practice, and research. Future studies are needed to investigate more unique social factors that could impact quarantine and isolation, such as living status (i.e., living alone or with others, if family or friends are near, rural or urban environment). Community-based interventions, such as online counseling services and wellness programs, are required to reduce the pandemic’s negative impact and enhance the general population’s health and QOL [37]. The findings of this study provide baseline evidence and highlight the need to replicate the effects of the COVID-19 pandemic in larger samples of individuals with various chronic diseases.

## 5. Conclusions

Social isolation has significantly influenced various aspects of individuals’ quality of life (QOL) and physical and mental health. This study investigated the effect of social isolation on QOL during the COVID-19 pandemic in Saudi Arabia. Recent research concluded that both physical and mental health statuses were affected by social isolation during the COVID-19 pandemic in Saudi Arabia. In addition, the results demonstrated that partially socially isolated participants appeared to have significantly better physical health status than completely socially isolated participants.

## Figures and Tables

**Table 1:** Sociodemographic and clinical characteristics of the studied participants (N = 775).

Variables	N	%
Gender		
Male	263	33.9
Female	512	67.2
Educational level		
Secondary	98	12.6
High school	164	21.2
College and above	513	66.2
Employment status		
Government employee	308	39.7
Private sector employee	137	17.7
Unemployed	284	36.7
Free business	46	5.9
Presence of chronic diseases		
Yes	146	18.8
No	629	81.2
Type of chronic disease		
Respiratory diseases such as asthma	44	30
Heart disease	9	6.2
Diabetes	51	34.9
Hypertension	37	25.4
Kidney failure	5	3.4
Isolation status		
Partial	682	88
Complete	93	12

**Table 2:** Mean score of the health-related quality-of-life short-form scale in the studied sample (N = 775).

SF-12 Items	Mean	SD
Health rating in general (GH1)	4.34	0.800
Limitation in moderate activities (PF02)	2.50	0.686
Limitation in climbing stairs (PF03)	2.58	0.623
Accomplished less due to emotional problems (RE2)	0.40	0.490
Not as careful due to emotional problems (RE3)	0.37	0.484
Accomplished less due to physical health (RP2)	0.24	0.427
Limited in the kind of work due to physical health (RP3)	0.22	0.414
Interference with social activities due to physical or emotional health (SF2)	3.05	1.210
Had a lot of energy (VT2)	3.59	1.240
Interference of pain (BP2)	3.95	0.966
Felt calm and peaceful (MH3)	4.07	1.376
Felt down (MH4)	4.21	1.332
PCS-12	44.7	8.49
MCS-12	34.67	6.15

**Table 3:** Statistical differences between participants’ isolation statuses and physical health (N = 775).

Scale	Isolation Status	N	Mean	Std. Deviation	(T) Value	Sig. *p* Value
**Physical health composite scale**	Partial	682	44.96	5.95	3.08	0.008
	Complete	93	42.88	7.27		

**Table 4:** Statistical differences between participants’ isolation statuses and mental health (N = 775).

Scale	Isolation Status	N	Mean	Std. Deviation	(T) Value	Sig. *p* Value
**Mental health composite scale**	Partial	682	34.72	8.35	0.396	0.115
	Complete	93	34.31	9.46		

**Table 5:** Statistical differences between participants’ demographic characteristics and mental health (N = 775).

Items		N	Mental Health Composite			ANOVA or *t*-Test	
			Mean	±	SD	Test Value	*p*-Value
**Age**	**18–30**	267	33.82	±	8.95	3.35	0.007 *
	**31–39**	234	34.07	±	8.40		
	**40–49**	154	35.61	±	8.06		
	**50–59**	73	36.35	±	7.21		
	**60 and above**	41	37.73	±	8.34		
**Gender**	**Female**	521	33.88	±	8.27	2.25	0.114
	**Male**	254	36.29	±	8.71		
**Field of employment**	**Government employee**	308	35.47	±	8.28	5.187	0.001 *
	**Private sector employee**	137	32.65	±	8.23		
	**Unemployed**	284	34.35	±	8.50		
	**Free business**	46	37.29	±	9.34		
**Educational level**	**Secondary**	98	35.52	±	8.60539	0.648	0.524
	**High school**	164	34.31	±	9.37402		
	**College and above**	513	34.62	±	8.17405		
**Presence of chronic illness**	**No**	629	34.74	±	8.33	0.794	0.373
	**Yes**	149	34.38	±	9.15		

* = Significant at *p* < 0.05.

**Table 6:** Statistical differences between participants’ demographic characteristics and physical health (N = 775).

Items		N	Physical Health Composite			ANOVA or *t*-Test	
			Mean	±	SD	Test Value	*p*-Value
**Age**	**18–30**	267	44.95	±	6.13	2.17	0.07
	**31–39**	234	45.29	±	5.98		
	**40–49**	154	44.21	±	5.74		
	**50–59**	73	43.69	±	7.11		
	**60 and above**	41	43.01	±	6.59		
**Gender**	**Female**	521	44.48	±	6.22	2.50	0.134
	**Male**	254	45.17	±	5.99		
**Field of employment**	**Government employee**	308	44.72	±	6.35	0.425	0.735
	**Private sector employee**	137	45.19	±	5.43		
	**Unemployed**	284	44.47	±	6.27		
	**Free business**	46	44.65	±	6.11		
**Educational level**	**Secondary**	98	43.8904	±	6.00292	1.01	0.362
	**High school**	164	44.9125		6.97673		
	**College and above**	513	44.8029		5.89682		
**Presence of chronic illness**	**No**	629	45.17	±	5.84	6.696	0.010 *
	**Yes**	149	42.70	±	7.01		

* = Significant at *p* < 0.05.

## Data Availability

The data are available upon reasonable request. Deidentified participant data are available from the corresponding author.

## Supplementary Materials

The following supporting information can be downloaded at: https://www.mdpi.com/article/10.3390/ijerph19116808/s1, File S1: SF-12 Health Survey.

## References

[1] Zhang Y., Ma Z.F. (2020). Impact of the COVID-19 Pandemic on Mental Health and Quality of Life among Local Residents in Liaoning Province, China: A Cross-Sectional Study. Int. J. Environ. Res. Public Health.

[2] World Health Organization WHO Director-General’s Opening Remarks at the Media Briefing on COVID-19—11 March 2020. https://www.who.int/director-general/speeches/detail/who-director-general-s-opening-remarks-at-the-media-briefing-on-COVID-19---11-march-2020.

[3] World Health Organization (2021). Coronavirus Disease (COVID-19) Pandemic. https://www.who.int/emergencies/diseases/novel-coronavirus-2019.

[4] Saudi Center for Disease Prevention and Control (2020). (COVID-19) Disease Interactive Dashboard. https://COVID19.cdc.gov.sa/daily-updates/.

[5] Nussbaumer-Streit B., Mayr V., Dobrescu A.I., Chapman A., Persad E., Klerings I., Wagner G., Siebert U., Ledinger D., Zachariah C. (2020). Quarantine alone or in combination with other public health measures to control COVID-19: A rapid review. Cochrane Database Syst. Rev..

[6] Matias T., Dominski F.H., Marks D.F. (2020). Human needs in COVID-19 isolation. J. Health Psychol..

[7] Banerjee D., Rai M. (2020). Social isolation in COVID-19: The impact of loneliness. Int. J. Soc. Psychiatry.

[8] Center for Disease Control and Prevention (CDC) (2020). Coronavirus 2019 (COVID-19) Stress and Coping. https://www.cdc.gov/coronavirus/2019-nCoV/index.html.

[9] Güzel P., Yildiz K., Esentaş M., Zerengök D. (2020). “Know-How” to Spend Time in Home Isolation during COVID-19; Restrictions and Recreational Activities. Int. J. Psychol. Educ. Stud..

[10] McCann P., Mackie P., Conacher A. (2017). Scottish Public Health Network Social Isolation & Loneliness: What is the Scope for Public Health Action?. Alison McCann Phil Mackie..

[11] Victor C., Scambler S., Bond J., Bowling A. (2000). Being alone in later life: Loneliness, social isolation and living alone. Rev. Clin. Gerontol..

[12] Sanders R. (2020). COVID-19, Social Isolation and Loneliness. https://www.iriss.org.uk/resources/esss-outlines/COVID-19-social-isolation-and-loneliness.

[13] Wilder-Smith A., Freedman D.O. (2020). Isolation, quarantine, social distancing and community containment: Pivotal role for old-style public health measures in the novel coronavirus (2019-nCoV) outbreak. J. Travel Med..

[14] Xiang Y.-T., Yang Y., Li W., Zhang L., Zhang Q., Cheung T., Ng C.H. (2020). Timely mental health care for the 2019 novel coronavirus outbreak is urgently needed. Lancet Psychiatry.

[15] Brooks S.K., Webster R.K., Smith L.E., Woodland L., Wessely S., Greenberg N., Rubin G.J. (2020). The psychological impact of quarantine and how to reduce it: Rapid review of the evidence. Lancet.

[16] McCulloch A. (2001). Social environments and health: Cross sectional national survey. BMJ.

[17] Algahtani F.D., Hassan S.-U.-N., Alsaif B., Zrieq R. (2021). Assessment of the Quality of Life during COVID-19 Pandemic: A Cross-Sectional Survey from the Kingdom of Saudi Arabia. Int. J. Environ. Res. Public Health.

[18] Sepúlveda-Loyola W., Rodríguez-Sánchez I., Pérez-Rodríguez P., Ganz F., Torralba R., Oliveira D.V., Rodríguez-Mañas L. (2020). Impact of Social Isolation Due to COVID-19 on Health in Older People: Mental and Physical Effects and Recommendations. J. Nutr. Health Aging.

[19] Reed S.B., Crespo C.J., Harvey W., Andersen R.E. (2011). Social isolation and physical inactivity in older US adults: Results from the Third National Health and Nutrition Examination Survey. Eur. J. Sport Sci..

[20] Ferreira L.N., Pereira L.N., Brás M.D.F., Ilchuk K. (2021). Quality of life under the COVID-19 quarantine. Qual. Life Res..

[21] Pietrabissa G., Simpson S.G. (2020). Psychological Consequences of Social Isolation during COVID-19 Outbreak. Front. Psychol..

[22] De Sousa R.D., Rodrigues A.M., Gregório M.J., Branco J., Gouveia M.J., Canhão H., Dias S. (2017). Anxiety and Depression in the Portuguese Older Adults: Prevalence and Associated Factors. Front. Med..

[23] Ozemek C., Lavie C.J., Rognmo Ø. (2019). Global physical activity levels—Need for intervention. Prog. Cardiovasc. Dis..

[24] Stieg C. (2020). When it’s All too much, Here’s How to Quell Coronavirus Anxiety, According to Experts. https://www.cnbc.com/2020/03/13/how-to-stay-calm-amid-coronavirus-pandemic-anxiety-relief-tips.html.

[25] Samlani Z., Lemfadli Y., Errami A.A., Oubaha S., Krati K. (2020). The impact of the COVID-19 pandemic on quality of life and well-being in Morocco. Arch. Community Med. Public Health.

[26] Vitorino L.M., Júnior G.H.Y., Gonzaga G., Dias I.F., Ribeiro I.M.G., Pereira J.P.L., França A.B., Al-Zaben F., Koenig H., Trzesniak C. (2020). Impact of social isolation strategies due to COVID-19 on mental health and quality of life in Brazil. SSRN.

[27] Chan J.F.-W., Yuan S., Kok K.-H., To K.K.-W., Chu H., Yang J., Xing F., Liu J., Yip C.C.-Y., Poon R.W.-S. (2020). A familial cluster of pneumonia associated with the 2019 novel coronavirus indicating person-to-person transmission: A study of a family cluster. Lancet.

[28] Huang C., Wang Y., Li X., Ren L., Zhao J., Hu Y., Zhang L., Fan G., Xu J., Gu X. (2020). Clinical features of patients infected with 2019 novel coronavirus in Wuhan, China. Lancet.

[29] Leigh-Hunt N., Bagguley D., Bash K., Turner V., Turnbull S., Valtorta N., Caan W. (2017). An overview of systematic reviews on the public health consequences of social isolation and loneliness. Public Health.

[30] Barnes M., Blom A.G., Cox K. (2006). The Social Exclusion of Older People: Evidence from the First Wave of the English Longitudinal Study of Ageing (ELSA), Final Report. https://www.ifs.org.uk/docs/odpm_social_exclusion.pdf.

[31] Szczepańska A., Pietrzyka K. (2021). The COVID-19 epidemic in Poland and its influence on the quality of life of university students (young adults) in the context of restricted access to public spaces. J. Public Health.

[32] De Belvis A.G., Avolio M., Sicuro L., Rosano A., Latini E., Damiani G., Ricciardi W. (2008). Social relationships and HRQL: A cross-sectional survey among older Italian adults. BMC Public Health.

[33] Al-Shehri A.H., Taha A.Z., Bahnassy A.A., Salah M. (2008). Health-related quality of life in type 2 diabetic patients. Ann. Saudi Med..

[34] Ware J.E., Kosinski M., Keller S.D. (1993). SF-12: How to Score the SF-12 Physical and Mental Health Summmary Scales.

[35] Goethals L., Barth N., Guyot J., Hupin D., Celarier T., Bongue B. (2020). Impact of Home Quarantine on Physical Activity Among Older Adults Living at Home During the COVID-19 Pandemic: Qualitative Interview Study. JMIR Aging.

[36] Lim S.L., Woo K.L., Lim E., Ng F., Chan M.Y., Gandhi M. (2020). Impact of COVID-19 on health-related quality of life in patients with cardiovascular disease: A multi-ethnic Asian study. Health Qual. Life Outcomes.

[37] Abdelbasset W.K., Alsubaie S.F., Tantawy S.A., Elyazed T.I.A., Elshehawy A.A. (2019). A cross-sectional study on the correlation between physical activity levels and health-related quality of life in community-dwelling middle-aged and older adults. Medicine.

